# Distal sagittal forelimb conformation in young Walloon horses: Radiographic assessment and its relationship with osteochondral fragments

**DOI:** 10.1371/journal.pone.0311965

**Published:** 2024-10-11

**Authors:** Raphaël Van Cauter, Isabelle Caudron, Jean-Philippe Lejeune, Alycia Rousset, Didier Serteyn

**Affiliations:** 1 Centre Européen du Cheval, Mont-le-Soie, Yvré-l’Évêque, Vielsalm; 2 Département des Sciences Cliniques des Équidés, Chirurgie et Orthopédie, FARAH, Université de Liège, Liège, Belgium; Universidade de Trás-os-Montes e Alto Douro: Universidade de Tras-os-Montes e Alto Douro, PORTUGAL

## Abstract

Osteochondral fragments within equine joints are commonly encountered and may predispose to lameness and limitation to sport purposes. Factors leading to this condition include genetic, nutritional and environmental conditions. However, few studies have evaluated the impact of conformation traits and their correlation with osteochondrosis. This study, based on the radiographic screenings of young horses born in Wallonia (266 individuals, 532 forelimbs), evaluated the correlation between foot, fetlock conformations of the front limb, height at the withers and presence of osteochondral fragments. Moreover, for all traits significantly associated with the presence of osteochondral fragments, a Receiver Operator Characteristic (ROC) curve, area under the curve and optimal cut-off value were calculated to predict the occurrence of fragments. Mean dorsal hoof wall angle was 52.36°, dorsal and palmar angle of the third phalanx were respectively 49.83° and 2.99°, and dorsal metacarpophalangeal angle 147.99°. Moreover, the prevalence of upright feet, defined as having an inclined profile of >2° steeper in relation to its contralateral counterpart, was 24%. Increased palmar angle of the distal phalanx was significantly correlated (*P* < 0.05) with presence of fragments located at the dorso-proximal margin of the proximal phalanx. The associated area under the curve was 0.623 (95% CI: 0528–0.717, *P* < 0.05) and the optimal cut-off value to predict fragment occurrence was 2.95° (sensitivity 77.3%; specificity 52.9%). Furthermore, the third metacarpal bone diameter of the left forelimb and height at the withers were significantly (*P* < 0.05) correlated with the presence of osteochondral fragments in general and within tarsocrural and metatarsophalangeal joints specifically. The area under the curve was 0.585 (95% CI: 0.513–0.656, *P* < 0.05) with an optimal cut-off value of 152.5 cm (sensitivity 85.1%; specificity 31.2%) for height at the withers to predict presence of osteochondral fragment; to predict the occurrence of osteochondral fragment in any joint on the basis of the third metacarpal bone diameter, the area under the curve was 0.595 (95% CI: 0.524–0.667, *P* <0.05) and the optimal cut-off value 34.9 mm (sensitivity 52.5%; specificity 64.9%). This study provides information about phenotypic traits associated with osteochondral fragments in horses. Although the diagnostic accuracy of these traits to detect osteochondral fragment was limited, the identification of more phenotypic characteristics could, in the future, make it possible to generate models for accurately identifying individuals at high risk of osteochondral fragments on the basis of their phenotype.

## Introduction

Orthopaedic pathologies are one of the main causes of retirement in racehorses and sport horses [1–3]. Among these, osteoarticular conditions are frequently the cause of lameness. In sport horses, the metacarpophalangeal joint is particularly affected [4–6]. The conformation of the distal extremity and its influence on pathologies such as navicular disease or suspensory ligament desmitis have been the subject of numerous studies in recent years [7–12].

The presence of conformational abnormalities impacts the horse’s movement and joint loads, predisposing horses to lameness and early retirement from competition [3,7,9,13–15]. Foot balance therefore plays a central role in the prevention of musculoskeletal injuries in the horse [4,15,16]. While raising the heels increases the loads on the suspensory apparatus and the extension of the metacarpophalangeal joint [17–20], raising the toe, on the other hand, puts more strain on the podotrochlear apparatus and the deep digital flexor tendon [17,21].

Among osteoarticular pathologies, osteochondrosis dissecans (OCD), a developmental orthopaedic pathology characterised by a defect in enchondral ossification, is common in equids [22–25]. Although a large proportion of affected horses do not present clinical signs, OCD can lead to the appearance of cartilage lesions, osteoarthritis and, ultimately, lameness, which has an impact on the individual’s sporting career [26–28]. The origin of OCD is multifactorial. The prevalence and dynamics of the condition appear to be influenced by genetic, environmental and dietary factors [24,29–32]. For the purposes of this article, all osteochondral fragments (OF), regardless of their origin, will be referred to as OF [33,34]. Generally speaking, OFs develop in regions subjected to compression and shear forces, so it seems that a biomechanical component plays a role in their development [25,35,36].

While in humans and pigs, the impact of certain conformational characteristics on the development of OCD has been demonstrated [37,38], in horses, few studies have identified correlations between morphological traits and OCD. A study by Bergmann and colleagues demonstrated that the presence of osteochondrosis within cervical facet joints was associated with the morphology of the joint concerned [39]. However, to our knowledge, only a correlation between a large cannon and the presence of OFs has been found at the appendicular level [40–42]. Given the biomechanical component of the development of OFs, various conformational traits that alter the forces to which the joints are subjected should be evaluated in order to identify individuals and joints at risk of developing OFs.

The aim of this study is to characterize the conformation of the distal extremity of young Walloon sport horses and to determine whether there is a correlation between certain conformational features of the foot and fetlock and the presence of OF by radiographic examination. Several hypotheses will therefore be verified:

- The presence of a correlation between metacarpophalangeal OF and verticalization of foot and distal phalanx angles
- The correlation between the presence of metacarpophalangeal OF and a low dorsal metacarpophalangeal joint angle (DMCPA)
- An increased prevalence of various OF lesions in individuals with a large diameter of the third metacarpal bone (DMCIII) and height at the withers.

## Materials and methods

### Population

The individuals were presented on the basis of a call for applications as part of a screening programme for developmental orthopaedic pathologies promoted by the Walloon Region [43].

This programme was aimed at sport horse breeders in the Walloon region and gave them the opportunity to carry out radiographic examinations on foals from birth to 36 months. A document describing the procedure and objectives of the programme was given to each animal owner and to the treating veterinarian for approval before the individuals were examined. These examinations were carried out at the Centre européen du Cheval de Mont-le-Soie or at the breeder’s premises. All foals from the same farm were systematically examined and their height at the withers was measured within the limits of their cooperation.

### Radiographic examinations

Radiographic examination of all animals included the following views: latero-medial of the 4 fetlocks, latero-medial and plantarolateral-dorsomedial oblique of the hocks, latero-medial (or slightly caudolateral-craniomedial oblique) of the stifles and latero-medial of the forefeet. All X-rays were taken on a flat, even surface; to take the views of the front feet and fetlocks, the animal stood squarely.

For radiographic images of the feet, after cleaning if necessary, the horses were placed with their front feet on 7 cm-high wooden rectangular blocks. To optimise radiographic examinations, the animals were sedated with romifidine hydrochloride (0.04mg/kg IV) or detomidine hydrochloride (0.01mg/kg IV) in combination with Butorphanol (0.02mg/kg IV). Additional views were performed when deemed necessary by the operator.

X-ray images were generated using a Gierth RHF 200 ML portable X-ray machine.

From 2016 to 2020, radiographs were developed with an Examion CR Vita 45 scanner and reviewed in digital format with Vita CR System Software V.3.2 (Carestream Health Rochester, NY, USA).

From 2021 to 2023, the radiographs were taken with the FUJIFILM Console Advance Software and sent to the VSOL programme for storage and reading.

### Radiographic interpretation & measures interpretation

Radiographs were examined for the presence of lesions by I.C. and measurements were taken independently by R.V.C. without knowledge of each other’s results. All forms of OF described in the literature were analysed. An OF was considered to be present when a fragment was observed adjacent to a joint margin. Variations in the appearance of the distal end of the medial trochlear ridge of the talus ("dewdrop lesion") were considered to be anatomical variations and not lesions [44].

**Fig 1:**
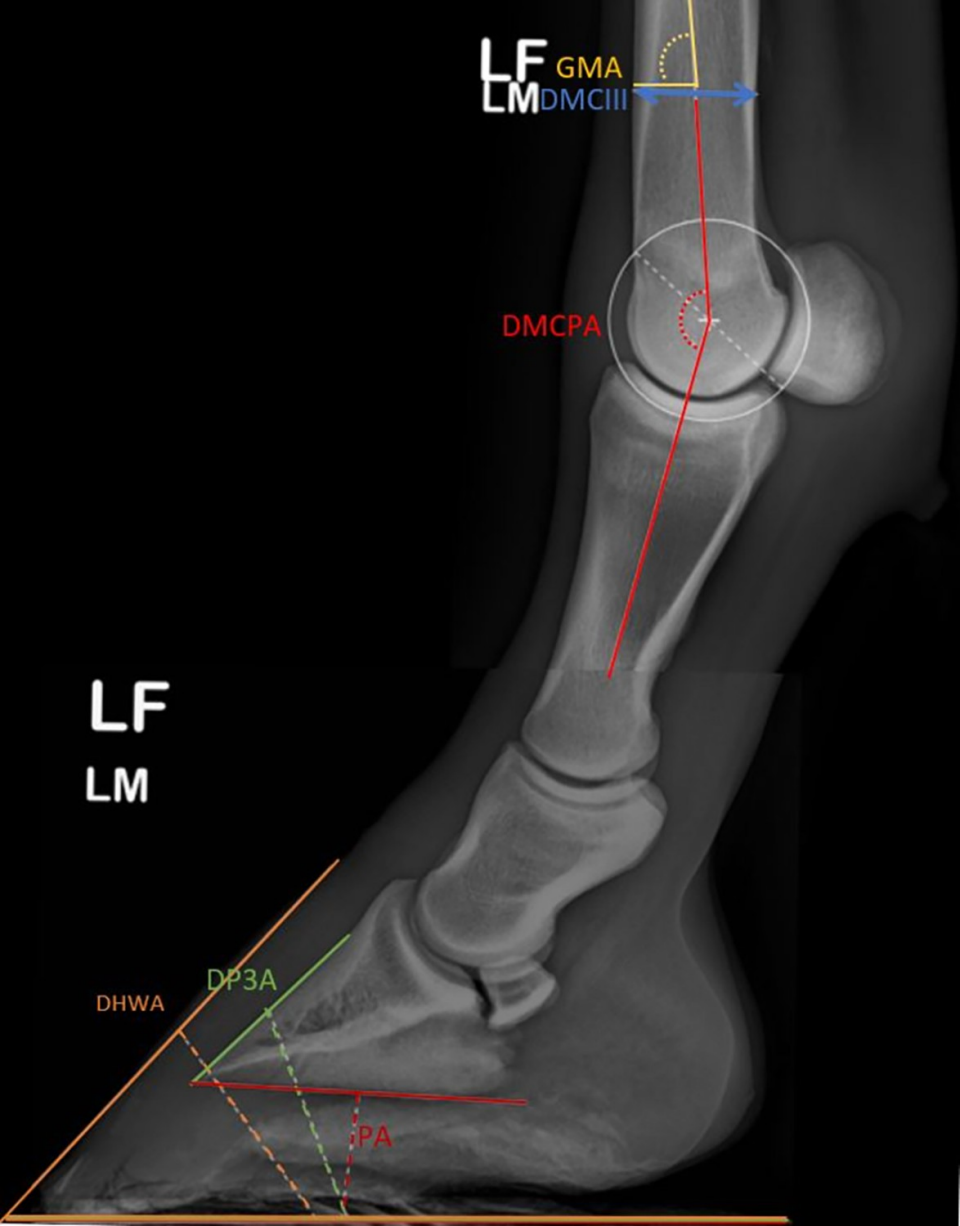
Latero-medial radiographs of an individual’s left foot and fetlock, showing the different measurements performed. GMA: Ground to third Metacarpal bone Angle; DMCIII: Diameter of the third Metacarpal bone; DMCPA: Dorsal Metacarpophalangeal Angle; DHWA: Dorsal Hoof Wall Angle; DP3A: Dorsal Third Phalanx Angle; PA: Palmar Angle.

The following parameters were measured by R.V.C.: The DMCIII, DMCPA, angle between the solar surface of the third phalanx and the ground (PA), angle between the dorsal surface of the third phalanx and the ground (DP3A), angle between the dorsal surface of the hoof wall and the ground (DHWA) and angle between the ground and the long axis of the third metacarpal bone (GMA) (reference points for each measurement are given in S1 Table) (Fig 1). In order to minimize the risk of error associated with measurements, they were repeated twice at a time interval greater than two weeks, except for GMA, which was carried out only once. Both values were averaged and used for statistical analysis. Each foot was classified as "upright" if the DHWA was >2° in relation to the contra-lateral limb of the same individual.

### Statistical analysis

Statistical analysis was performed using Microsoft Excel software and XLSTAT (Assinsoft, Paris, France) with a significance level <0.05. The Shapiro-Wilk test was used to determine the normality of data distribution and the χ2/Fisher’s exact test to compare the variance of the samples. Each joint site, each joint and each animal was classified on a binary scale where 0 represented the absence of a fragment and 1 its presence.

### Correlation between conformation parameters, height and age

To assess the correlation between the different linear variables measured, a Pearson correlation test was carried out for all measurements whose distribution followed a normal distribution and a Spearman correlation coefficient was calculated in the opposite case. The correlation calculated was then interpreted as follows: r<0.1- negligible correlation, r = 0.1–0.39—weak correlation, r = 0.4–0.69—moderate correlation, r = 0.7–0.89—strong correlation, r = 0.9–1.0—very strong correlation [45]. DMCPA was correlated for each limb with DHWA, DP3A, PA, GMA and DMCIII. In addition, for each individual, a correlation matrix was produced based on age, height and the mean of DHWA, DP3A, PA and DMCIII of the two distal extremities of the forelimbs and interpreted as described above.

### Correlation between the presence of OF and the conformation of the distal extremity

To determine whether there was a significant correlation between the various parameters measured and the presence of OF, a one-tailed independent t-test or Mann-Withney (if the distribution of the variables did not follow a normal distribution or if the ratio between the variances of the samples was different from 1) was carried out.

In order to determine whether there was an association between OF presence in individuals and high height or large DMCIII, a one-tailed unpaired t-test or Mann-Withney-if the samples did not follow a normal distribution or the ratio between the variances was equal to one-was carried out on all individuals for whom these two parameters (height and left DMCIII) had been assessed. The left DMCIII was chosen as the reference limb in order to be consistent with previously published data [40,41].

For all parameters analysed that were significantly correlated with the presence of OFs, receiver operating characteristic (ROC) analysis was used to estimate the predictive value of the parameter analysed on the development of OFs with which it was significantly correlated.

A one-tailed Z-test was performed to assess whether the proportion of upright feet was significantly higher when a fragment was present within the limb.

The repeatability of the DHWA, DP3A, PA, DMCPA and DMCIII measurements was assessed using Lin’s Concordance Correlation Test [46,47]. The Lin’s concordance correlation coefficient obtained was interpreted as follows: < 0.50 Unacceptable, 0.51–0.60 Poor, 0.61–0.70 Mediocre, 0.71–0.80 Satisfactory, 0.81–0.90 Fairly good, 0.91–0.95 Very good, > 0.95 Excellent [48].

## Results

A group of 266 individuals was included in the study. The gender distribution followed a perfect parity (133 individuals of each sex). The mean age at examination was 651 days (SD: 67.2; Max: 906; Min: 473 days). The distribution of animals within the different studbooks was as follows: 142 SBS, 38 Zangersheide, 19 Luxembourg Saddlebred, 17 Belgische Warmblood Paard, 14 Arabian, 12 Hanoverian, 11 AngloEuropean, 8 Selle Français, 4 Pura Raza Española and 1 Quarter-Horse. The height of 255 individuals was assessed, the average being 156 cm (SD: 5.8; Max: 173 cm; Min. 131 cm).

### Distal extremity conformation

The mean DHWA, DP3A and PA angles were 52.36°(SD: 3.52; Max: 65.7°; Min: 42.65°), 49.83° (SD: 3.6; Max: 62.85°; Min: 39.7°) and 2.99° (SD: 3.57; Min: 11.1°; Min: -6.5°), respectively.

The mean DMCPA was 147.99° (SD: 4.6; Min: 135.7°; Max: 168.2°) and the mean DMCIII was 34.35 mm (SD: 2.7; Max: 45.4; Min: 24.2).

The correlation between the DMCPA and the different measurements for each distal extremity was weak and values are shown in Table 1.

**Table 1:** Correlation between DMCPA and DHWA, DP3A, PA, GMA and DMCIII for each distal extremity.

Parameter:	DMCPA
	Corr coef (r)
DHWA	**-0,15**
DP3A	**-0,12**
PA	**-0,25**
GMA	**0,194**
DMCIII	**0,113**

DMCPA: Dorsal Metacarpophalangeal Angle; DHWA: Dorsal Hoof Wall Angle; DP3A: Dorsal Third Phalanx Angle; PA: Palmar angle of the third phalanx; GMA: Angle between ground surface and third Metacarpal bone; DMCIII: Diameter of the Third Metacarpal Bone. N = 532. **In Bold: Significant correlation (p<0.05).**

The interaction between age, height and mean measurements of the two distal extremities of the forelimbs of individuals for which all these parameters were available (n = 255) ranged from 0.657 to 0.004. Significant correlation coefficients (p<0.05) were present between: height and age (r = 0.208); height and DMCIII (r = 0.657); DMCPA and height (r = 0. 171); DMCIII and DMCPA (r = 0.125); DMCPA and DHWA (r = -0.164); DMCPA and DP3 (-0.130); DMCPA and PA (-0.279); DHWA and DP3A (r = 0.895) and DHWA and PA (r = 0.623) (Table 2).

**Table 2:** Correlation matrix including interactions between age, height at the withers and distal extremity conformation in 255 young horses.

Variables	age	height	DMCIII	DMCPA	DHWA	DP3A	PA
age	**1**	**0,208**	-0,074	0,047	-0,096	-0,059	-0,008
height	**0,208**	**1**	**0,657**	**0,171**	0,034	-0,004	-0,033
DMCIII	-0,074	**0,657**	**1**	**0,125**	-0,034	-0,04	-0,082
DMCPA	0,047	**0,171**	**0,125**	**1**	**-0,164**	**-0,130**	**-0,279**
DHWA	-0,096	0,034	-0,034	**-0,164**	**1**	**0,895**	**0,623**
DP3	-0,059	-0,004	-0,040	**-0,130**	**0,895**	**1**	**0,690**
PA	-0,008	-0,033	-0,082	**-0,279**	**0,623**	**0,690**	**1**

DMCIII: Diameter of the left third Metacarpal Bone; DMCPA: Dorsal metacarpophalangeal angle; DHWA: Dorsal Hoof Wall Angle; DP3A: Dorsal Third Phalanx Angle; PA: Palmar angle of the third phalanx. N = 255. **In Bold: Significant correlation (p<0.05).**

The repeatability of measurements, assessed by Lin’s concordance correlation coefficient, ranged from fairly good to excellent, with values of 0.892 (95% CI: 0.874–0.908) for DMCIII, 0. 931 (95% CI: 0.919–0.942) for DMCPA, 0.962 (95% CI: 0.954–0.967) for PA, 0.976 (95% CI: .971–0.98) for DHWA and 0.981 (95% CI: 0.977–0.984) for DP3A.

### Prevalence of OFs

A group of 106 individuals (39.8%) had at least one OF. The joint most affected was the metatarsophalangeal joint with 56 individuals concerned (21%), followed by the metacarpophalangeal joint (35 individuals; 13%), the tarsocrural joint (21 individuals; 7.9%), the femoropatellar joint (20 individuals; 7.5%) and the distal interphalangeal joint (5 individuals; 1.8%).

### Correlation between presence of OF and conformation of the distal extremity

With regard to the presence of an interaction between the angles measured and the presence of OF within the same limb, only the PA and the presence of OF at the dorso-proximal aspect of the first phalanx (DPP1) were correlated. The limbs with this type of fragment having a significantly higher PA than the group without (p = 0.021) (Table 3).

**Table 3:** Conformation of 532 front limb distal extremity (266 individuals) and interaction with the presence of metacarpophalangeal joint osteochondral fragments.

			DHWA	DP3A	PA	DMCPA	GMA	N
Fragment	DP P1	present	52,43	50,20	3,83	147,30	88,82	22
		absent	52,36	49,81	2,96	148,02	88,55	510
		p-value	0,428^b^	0,279^b^	**0,021^b^**	0,251^b^	0,760^a^	
	dorsal MCIII	present	51,49	48,59	2,96	147,86	88,34	18
		absent	52,39	49,87	3,00	147,99	88,57	514
		p-value	0,916^b^	0,932^a^	0,524^a^	0,572 ^b^	0,813^a^	
	POF	present	56,43	53,45	4,37	149,55	89,90	3
		absent	52,34	49,81	2,99	147,98	88,55	529
		p-value	0,081^b^	0,064^b^	0,182^a^	0,796^b^	0,569^a^	
	MCP	present	52,32	49,74	3,50	147,82	88,62	41
		absent	52,36	49,83	2,95	148,00	88,56	491
		p-value	0,654^b^	0,588^b^	0,098^a^	0,519^b^	0,929^a^	
Total			52,36	49,83	2,99	147,99	88,56	532

DHWA:Dorsal Hoof Wall Angle; DP3A: Dorsal Third Phalanx Angle; PA: Palmar Angle of the third phalanx; DMCPA: Dorsal Metacarpophalangeal Angle; GMA: Ground to third Metacarpal bone Angle; N: Number of distal extremity evaluated; DP P1: Dorso-proximal First phalanx; MCIII: Third metacarpal bone; POF: Palmar/plantar osteochondral fragment; MCP: Metacarpophalangeal joint; ^a^: T-test; ^b^: Mann-Withney test. **In Bold: Significant correlation (p<0.05)**

As regards to the presence of an interaction between an upright foot and OF within the same limb, no significant interaction could be identified out of the 532 limbs assessed (Table 4).

**Table 4:** Prevalence of front limb upright foot and osteochondral fragment within the same distal extremity.

			Upright foot	No upright foot	% upright foot	N
Fragment	P2-P3	present	2	3	40,00	5
		absent	128	399	24,29	527
		p-value	0,399^a^			
	DP P1	present	4	18	18,18	22
		absent	126	384	24,71	510
		p-value	0,689^a^			
	Dorsal MCIII	present	7	11	38,89	18
		absent	123	391	23,93	514
		p-value	0,15^a^			
	POF	present	2	1	66,67	3
		absent	128	401	24,20	529
		p-value	0,173^a^			
	MCP	present	13	28	31,71	41
		absent	117	374	23,83	491
		p-value	0,192^a^			
Total			130	402	24,44	532

N = number of distal extremity evaluated; P2-P3: Distal interphalangeal joint; DP P1: Dorso-proximal First phalanx; MCIII: Metacarpal III bone; POF: Palmar/plantar osteochondral fragment; MCP: Metacarpophalangeal joint; ^a^: Z-test. **In Bold: Significant correlation (p<0.05).**

Finally, the presence of a correlation between left DMCIII or height at the withers and OF within the different joints was also assessed. Individuals with one or more OFs in the metatarsophalangeal, tarsocrural or at least one joint had a significantly larger left DMCIII (Table 5). Relatively similar results were observed for the presence of OFs and the size of the individuals. Individuals with an OF in the distal interphalangeal, metatarsophalangeal, tarsocrural or at least one joint were significantly taller on examination than those without OF.

**Table 5:** Prevalence of osteochondral fragment and relation with the diameter of the left front third metacarpal bone and height at the withers of 255 young horses.

Fragment	Location		MCIIID LF	height	N
	P2-P3	present	35,95	164,50	2
		absent	34,30	156,00	253
		p-value	0,15**^b^**	**0,007^b^**	
	MCP	present	34,55	155,47	34
		absent	34,27	156,16	221
		p-value	0,269**^b^**	0,728**^b^**	
	MTP	present	34,83	157,60	53
		absent	34,17	155,67	202
		p-value	**0,015^b^**	**0,019^b^**	
	TC	present	35,69	159,14	21
		absent	34,19	155,79	234
		p-value	**0,013^b^**	**0,006^b^**	
	FP	present	34,92	157,06	17
		absent	34,27	156,00	238
		p-value	0,138^b^	0,232^b^	
	Total	present	34,83	157,06	101
		absent	33,97	155,42	154
		p-value	**0,005^b^**	**0,011^b^**	
overall population			34,31	156,07	255

MCIIID LF: Diameter of the Third metacarpal bone of the left front limb; N = Number of individuals included; P2-P3: Distal interphalangeal joint; MCP:Metacarpophalangeal joint; MTP: Metatarsophalangeal joint; TC: Tarsocrural joint; FP: Femoro-patellar joint

^b^: Mann-Whitney test. **In Bold: Significant correlation (p<0.05).**

### Predictive value of conformational traits on the development of OFs

**Fig 2:**
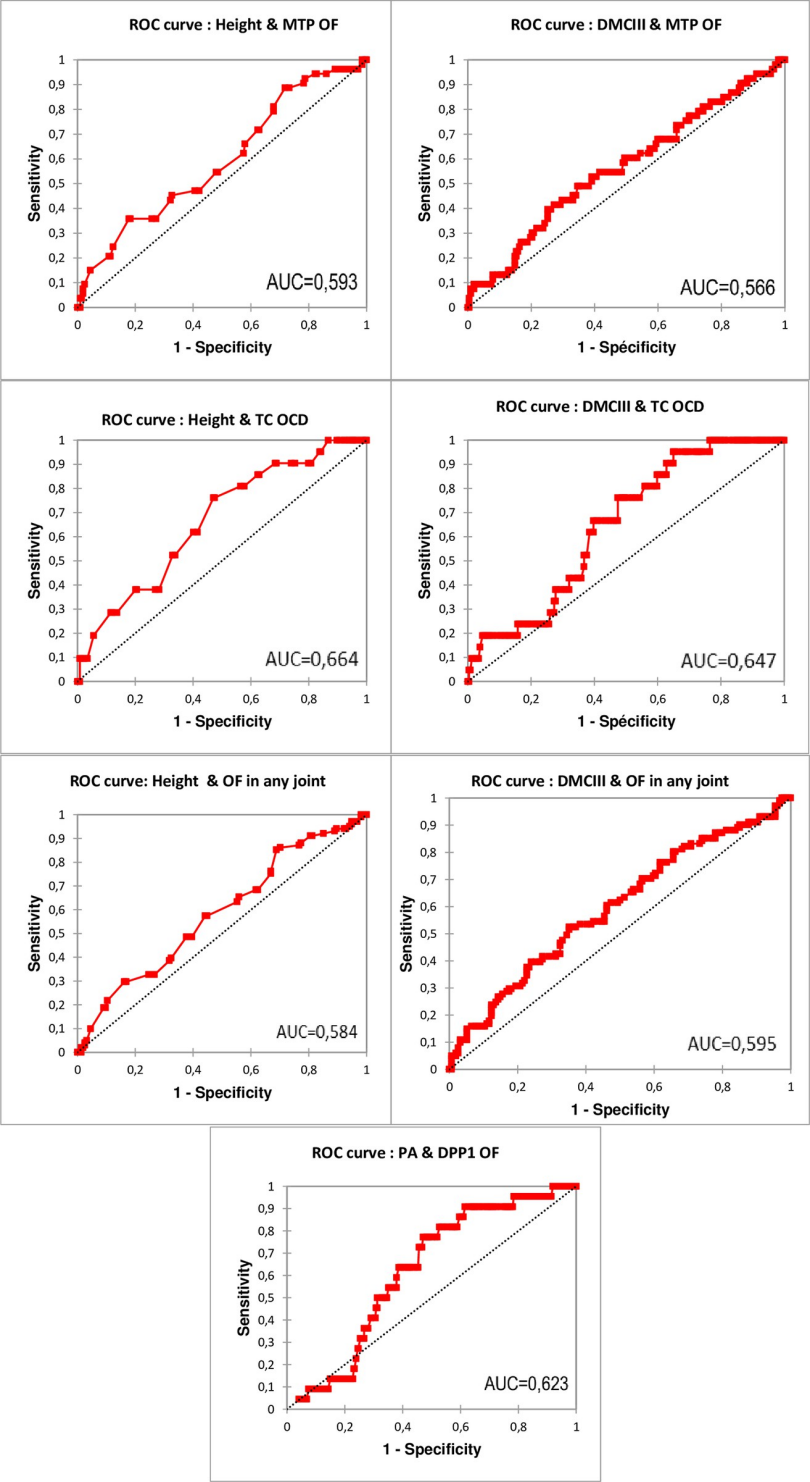
ROC curve and area under the curve according to different morphological traits significantly associated with osteochondral fragment at different locations. MTP: Metatarsophalangeal joint; OF: Osteochondral fragment; DMCIII: Diameter of the third metacarpal bone; TC: Tarsocrural; OCD: Osteochondrosis dissecans; PA: Palmar Angle of third phalanx; DPP1: Dorso-proximal First Phalanx.

ROC curve was generated to evaluate the prognostic value of the PA for DPP1 OF in the limb. The area under the curve was 0.623 (95% CI: 0.528–0.717, *p* < 0.05), the optimal cut-off value of the PA in predicting DPP1 OF was 2.95°, with a sensitivity of 77.3% and a specificity of 52.9% (Fig 2).

The ROC curve for the height and DMCIII to predict the presence of OF in the tarsocrural, metatarsophalangeal or any joint are given in Fig 2. The area under the curve for height was 0.593 (95% CI: 0.506–0.679, *P* < 0.05) to predict metatarsophalangeal OF, 0.664 (95% CI: 0.549–0.780, *P* < 0.05) for tarsocrural OCD and 0.585 (95% CI: 0.513–0.656, *P* < 0.05) for OF in any joint. Considering DMCIII to predict OF in metatarsophalangeal, tarsocrural or OF in any joint, the area under the curve were respectively 0.566 (95% CI: 0.476–0.655, *P* < 0.05), 0.647 (95% CI: 0.545–0.749, *P* < 0.05) and 0.595 (95% CI: 0.524–0.667, *P* <0.05). The optimal cut-off value for height at the withers and DMCIII to predict MTP and TC OF or OF in any joint and the associated sensitivity and specificity are given in Table 6.

**Table 6:** Optimal cut-off value and associated sensitivity and specificity of height at the withers and left front third metacarpal bone diameter to predict osteochondral fragment in tarsocrural, metatarsophalangeal or in any joint.

Traits	OF location	Optimal cut-off Value	Sensitivity	Specificity
Height	TC	156,5 cm	0,762	0,53
	MTP	160,5 cm	0,358	0,822
	Overall	152,5 cm	0,851	0,312
DMCIII	TC	33,05 mm	0,952	0,35
	MTP	35,1 mm	0,491	0,653
	Overall	34,9 mm	0,525	0,649

OF: Osteochondral Fragment; TC: Tarsocrural joint; MTP: Metatarsophalangeal joint; Overall: Any joint examined; DMCIII: Diameter of the third metacarpal bone

## Discussion

### Distal extremity conformation

The results of the present study are consistent with a number of publications. Indeed, previous studies have evaluated the effect of artificial elevation of the toe or heels on leg forces and angles up to the elbow [18–20,49–51]. This shows that the more distal the segment under consideration, the greater the impact of a change in foot angle on joint forces and angles [18,20,50,51]. In addition, recent publications demonstrate that an artificial increase in heel height induces extension and an increase in the loads exerted on the metacarpophalangeal joint (and distal interphalangeal and proximal interphalangeal flexion) following a linear relationship [18,20,49–52]. However, it seems that metacarpophalangeal extension during heel elevation follows a linear relationship up to a certain degree of heel elevation of around 15°, above which the fetlock flexes by around 7–8° per 10° of elevation [19]. With regard to the evaluation of conformation and the relationship between the foot and fetlock in a population of horses, Hagen & colleagues did not find any significant correlation between DMCPA and PA or DHWA in a study of 30 individuals [10].

In this study, including 266 horses, we demonstrated that DHWA, PA and DP3A are weakly but significantly correlated with DMCPA in an inversely proportional way, which translates into an increase in fetlock extension when the foot has a more vertical profile.

### Correlation between presence of OF and conformation of the distal extremity

In our study, the fact that a foot with a more upright profile (DHWA, DP3A or PA increase) seems to be weakly but significantly associated with fetlock extension during bipodal stance at rest could explain the correlation between this conformation and the presence of OF within the fetlock. Given that it has been demonstrated that, during the hyperextension phase of the metacarpophalangeal joint, particularly during rapid movement, the contact surface between the third metacarpal bone and the first phalanx increased with a shift in dorsal direction[53,54], an increase in the extension of the metacarpophalangeal joint increases pressures at the level of the contact zone between the DPP1 and the proximal aspect of the condyle of the third metacarpal bone. Thus, an increase in fetlock extension induces an increased impingement of the DPP1 with the dorsal aspect of the third metacarpal condyle during movement, resulting in greater compression and shear forces. These are considered to be a cause of the different fetlock OFs [25]. According to this hypothesis, a conformation that favors the extension of the metacarpophalangeal joint would lead to more stress being placed on joint areas susceptible to develop OFs, which correlates with the results of our study showing that the presence of DPP1 OF is significantly correlated with a higher PA and that there is a (non-significant) trend between a correlation of different angles of the foot (DHWA, DP3A, PA) and POFs despite only 3 individuals being affected.

In contrast to the above assumption, one hypothesis of the study presented here is that OFs induce verticalization of the foot. In fact, various studies have shown that the presence of chronic asymmetry during movement modifies the forces exerted on the hoof, causing straightening of the hoof wall and atrophy of the hoof [4,55]. On the other hand, the impact of the presence of osteochondrosis on the movement kinetics of foals is controversial. Indeed, studies have shown an influence, whereas Gorissen & colleagues, in a group of 10 individuals, were unable to observe any [56–58]. In our study, front limbs with one or more OFs lesions did not show significant hoof uprightness compared with the contralateral limb. This suggests that hoof uprightness is primary due to lesion development. However, with the exception of DPP1 OFs, a tendency towards a verticalized foot on the side of the lesion was observed and the absence of significant results could be linked to the size of the samples.

Another hypothesis we postulated was an increase in the prevalence of various OFs lesions in individuals with a wide DMCIII. The results obtained show a correlation between height at the withers, DMCIII and the presence of OFs. Since the individuals could not be examined at the same age, and since age influences the height of the individuals, the DMCIII was considered to be a more reliable parameter since it was not correlated with age (r = -0.074, not significant). The analysis shows that individuals with OFs are taller and have a wider DMCIII. Previous publications had already observed this interaction between the presence of tarsocrural OFs and a large cannon [40–42]. It is interesting to note that the heritability of OCD varies according to breed and joint and is highest in the hock [26,59,60]. From the many studies carried out, it appears that, among the Loci at risk, there is a strong association between OCD of the hock and a SNP on chromosome ECA 3 63 kb upstream to the LCORL gene (Ligand-dependant Receptor Corepression-like Gene) which is a gene whose expression is positively correlated with height in Hanoverian horses [61]. However, how individual height influences the risk of developing OFs is not yet clear, notably whether this results from higher biomechanical loads in taller individuals or is inherent to other characteristics correlated with height, such as the thickness of the epiphyseal growth cartilage and the number of vessels circulating in it [62].

Finaly, the results of the ROC curves and associated areas under the curve of the different traits for detecting the presence of fragments were significant, indicating that, based on these criteria taken individually, the probability of detecting individuals or limbs with these OFs was significantly higher than detecting affected individuals at random. However, in the context of diagnostic testing, in order to be considered clinically useful, the area under the curve must be greater than 0.80, which was not the case for any of the parameters tested [63]. In the future, it would be necessary to evaluate other phenotypic traits in order to generate complex models for accurately detecting individuals at high risk of developing OFs.

### Limitations

The study presented here has a number of limitations, in particular the lack of longitudinal follow-up of the individuals. In this study, the individuals were radiographed between 473 and 906 days of age. It has been shown that the conformation of the distal extremity changes during growth [13,58,64–66]. Furthermore, the evolutionary window of OFs is articulation-dependent [67–69]. It is therefore possible that certain conformational traits were present at critical moments and were no longer present at the time of examination.

Another inherent limitation of this study is that radiographic examination offers limited sensitivity in assessing osteochondrosis lesions [70–72]. In addition, the standard protocol applied included only lateromedial views of all the joints, with the exception of the hock. Thus, the absence of dorsopalmar views in the examinations made it impossible to assess variations in lateromedial conformation and their correlation with the presence of OFs. This protocol, as described in the study by Denoix & al., was used in order to comply with the ALARA radiation protection principle and to minimise the risk of accidents to operators when taking radiographs on foals that were often poorly handled [22]. In order to improve the sensitivity of the examination performed, ultrasound screening as described by Hoey 2022 or an increased number of radiographic views per joint would be indicated [69–71,73].

In addition, the population of horses studied here came from different farms in Wallonia, so the breeding method was not standardised. Consequently, certain factors that were not evaluated (type of ground and bedding, activity, frequency of trimming) may have influenced the appearance of OFs as well as conformation. The individuals came from different origins and breeds; although OFs in the joints studied have been described in the literature in all of these breeds, it is possible, as shown by the heterogeneity of heritability estimates from previous studies, that the proportion linked to the different factors varies according to breed [26,67,74,75].

Furthermore, Pauwels & colleagues have demonstrated an influence of the cranio-caudal position of the leg on the interphalangeal angles, and no effect on the palmar angle of the third phalanx. The study by Alrtib and colleagues on 5 horses also showed a modification of the DMCPA during unipodal versus bipodal stance, but no significant effect of the position of the head [76]. However, the authors of the present study feel that, despite very good measurement repeatability, the DMCPA is not a totally reliable measure, as the force exerted on each limb, even in bipodal stance, is not perfectly symmetrical and is subject to variation, particularly according to the position of the head and neck. Finally, although all the radiographs were taken under standardised conditions in order to limit the effect of magnification in particular, i.e. with the planar sensor in contact with the structure of interest and the X-ray source at a constant distance from the planar sensor, calibration was carried out automatically (0.15mm/pixel) in the absence of a radiopaque marker. As a result, the measurements taken were probably overestimated, albeit to a limited extent (of approximately 0.075 cm) [77].

## Conclusion

This study showed a correlation between the presence of certain conformational traits and OFs: limbs with higher PA may be predisposed to be affected by DPP1 OF. In addition, taller individuals and/or those with a wider DMCIII seem to develop OFs more frequently, particularly in the tarsocrural and metatarsophalangeal joints. Therefore, it seems that certain phenotypic traits, notably the height at the withers and diameter of the third metacarpal bone, are associated with the presence of osteochondral fragments and that they should be taken into account in the context of selection and future research.

## Supporting information

S1 Table Parameters of interest with their abbreviations and references points used. (ODS)

## Abbreviations

OCD: Osteochondrosis Dissecans
OF: Osteochondral Fragment
DMCPA: Dorsal Metacarpophalangeal Joint Angle
DMCIII: : Third Metacarpal bone Diameter
PA: Palmar Angle of the Third Phalanx
DP3A: Dorsal Third Phalanx Angle
DHWA: Dorsal Hoof Wall Angle
GMA: Ground to Third Metacarpal Bone Angle
DPP1: dorso-proximal aspect of the first phalanx
